# A-Kinase Anchoring Proteins in Cardiac Myocytes and Their Roles in Regulating Calcium Cycling

**DOI:** 10.3390/cells12030436

**Published:** 2023-01-28

**Authors:** Hariharan Subramanian, Viacheslav O. Nikolaev

**Affiliations:** 1 Institute of Experimental Cardiovascular Research, University Medical Center Hamburg-Eppendorf, 20246 Hamburg, Germany; 2 German Center for Cardiovascular Research (DZHK), Partner Site Hamburg/Kiel/Lübeck

**Keywords:** Calcium cycling, cAMP signaling, Protein Kinase A, A-kinase anchoring protein, Phospholamban, Ryanodine receptor, calcium channel, SERCA2a

## Abstract

The rate of calcium cycling and calcium transient amplitude are critical determinants for the efficient contraction and relaxation of the heart. Calcium-handling proteins in the cardiac myocyte are altered in heart failure, and restoring the proper function of those proteins is an effective potential therapeutic strategy. The calcium-handling proteins or their regulators are phosphorylated by a cAMP-dependent kinase (PKA), and thereby their activity is regulated. A-Kinase Anchoring Proteins (AKAPs) play a seminal role in orchestrating PKA and cAMP regulators in calcium handling and contractile machinery. This cAMP/PKA orchestration is crucial for the increased force and rate of contraction and relaxation of the heart in response to fight-or-flight. Knockout models and the few available preclinical models proved that the efficient targeting of AKAPs offers potential therapies tailor-made for improving defective calcium cycling. In this review, we highlight important studies that identified AKAPs and their regulatory roles in cardiac myocyte calcium cycling in health and disease.

## 1. Introduction

A-kinase anchoring proteins (AKAPs) are a class of unrelated scaffolding proteins that bind and confine Protein Kinase A (PKA) close to its substrates. AKAPs possess 14–18 amino acid-long amphipathic alpha-helices that dock regulatory subunits (RI or RII) of PKA and a targeting domain to localize in different subcellular compartments and orchestrate cAMP signalosomes comprising adenylate cyclase (AC), phosphodiesterase (PDE), phosphatase and also PKA substrates [1]. Several AKAPs were identified in the heart [2,3], and they perform a plethora of functions including but not limited to calcium cycling and contraction. In adult cardiac myocytes, where the cytosolic cAMP concentration is estimated to be 1.2 µM [4], how PKA is maintained in a quiescent state has been worth investigating in the last few decades. After many detailed investigations on cAMP and PKA compartmentation, AKAPs were regarded as the key scaffolding molecules that confine PKA in microdomains guarded by PDEs that degrade and maintain cAMP as low as 100 nM [5]. AKAPs are important regulators of PKA, a broad range serine/threonine kinase that performs vastly different functions depending on the external stimuli.

In the heart, membrane depolarization- or excitation-induced calcium current or influx via L-type calcium channels (LTCC; Cav1.2) triggers large calcium-induced calcium release (CICR) from the sarcoplasmic reticulum (SR) via the major calcium release channel Ryanodine Receptor 2 (RyR2). The dramatic increase in calcium triggers myofilament contraction. For relaxation, most of the calcium is taken back into SR via the sarcoplasmic reticulum Ca2+ ATPase 2a (SERCA2a), and the SR is filled with calcium and is ready for the next contraction. The submembrane sodium-calcium exchanger (NCX) plays a small yet significant role in reducing excess cytosolic calcium in the cell. The proportion of calcium handled by NCX varies between species [6]. This is a recurrent calcium cycling process that occurs in a healthy heart.

**Figure 1:**
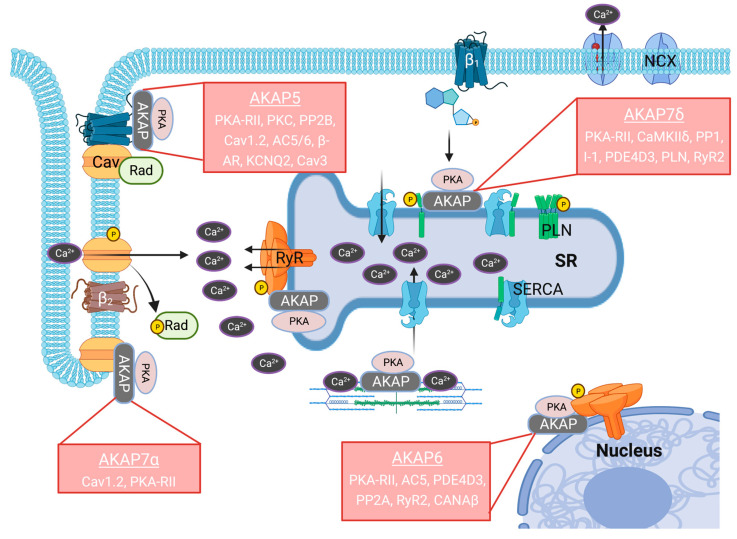
Calcium cycling in cardiac myocytes: depolarizing calcium currents via Cav1.2 induce CICR from SR via RyR2. This increase in cytosolic calcium increases the force of myofilament contraction. For relaxation, most of the calcium is taken back to the SR by SERCA2a, and a significant portion exits via NCX. The calcium-handling proteins Cav1.2, RyR2, and PLN are regulated by AKAP-bound PKA after β-adrenergic receptor (β_1_ and β_2_) stimulation. Different AKAPs regulating the calcium-handling proteins are mentioned. Though AKAPs bind multiple proteins, only the binding partners that play a role in calcium cycling are listed. Illustrations in Figure 1 are created with BioRender.com.

During stress or exercise, norepinephrine from sympathetic fibers and epinephrine from adrenals activate β-adrenergic receptor signaling in cardiac myocytes to increase the blood pumping force (inotropy), relaxation (lusitropy), and rate (chronotropy) of the heart. The increased inotropic, lusitropic, and chronotropic effects of the heart are mediated by cAMP increases in the microdomains and subsequent PKA activation and phosphorylation of substrates that regulate excitation–contraction coupling (ECC) [6]. Pioneering work from many laboratories revealed that PKA phosphorylates the following proteins involved in ECC. 1. Rad, a small RGK G-protein, which relieves the inhibition on Cav1.2 to augment calcium currents [7,8,9], 2. RyR2, which increases the open probability of the channel [10], 3. Phospholamban (PLN) to relieve the inhibition of SERCA2a [11], 4. Troponin I (TnI), a subunit of the troponin complex that promotes relaxation [12,13], 5. MyBPc3, a sarcomeric protein that increases contraction and relaxation [14], and 6. IK_S_, sarcolemmal potassium channel that accelerates repolarization [15] (Figure 1).

Although almost 12 different AKAPs were identified in the heart [2,3], only a handful of AKAPs were intensively studied for their role in calcium cycling and ECC. In this review, we would like to highlight the studies on AKAPs that regulate calcium cycling and ECC in physiological and pathophysiological settings. We also discuss the possible future directions for studies on AKAPs and how AKAPs can be used as therapeutic tools to improve calcium cycling and thereby cardiac function.

## 2. Importance of PKA Anchoring in Calcium Cycling and Contractility

So far, AKAP7, AKAP5, and AKAP6 have been identified to bind calcium-handling proteins in cardiac myocytes. Using the Ht31 peptide, a 24-amino acid peptide derived from the RII binding domain of AKAP-LBC (AKAP13) to disrupt the association between PKA-RII and AKAP is a classic way to identify the importance of AKAPs in any cellular context. The Ht31 peptide results in the delocalization of PKA-RII from the periodic striation and perinuclear region in myocytes. Surprisingly this leads to increased β-adrenergic-stimulated contraction and accelerated relaxation time in isolated cells. The authors explained the potential role of reduced TnI and MyBPC phosphorylation in Ht31-expressing cells for increased contraction and faster relaxation [16]. The same group also published the only available in vivo data on PKA disruption in the heart. Adenovirus-mediated Ht31 overexpression in the myocardium did not impair cardiac function under basal conditions. However, after acute isoproterenol treatment, they found increased inotropic responses in Ht31-expressing hearts and reduced phosphorylation of PKA substrates including RyR2 and PLN compared to the control [17]. Conversely, peptides derived from the PKA-binding region of AKAP10 to disrupt both PKA-RI and RII from AKAPs showed a pronounced effect of PKA disruption from AKAPs on contractility. In isolated cardiac myocytes, these peptides reduced isoproterenol-induced PLN and TnI phosphorylation and thereby reduced contraction and increased the time to contract and relax. In Langerndorff-perfused hearts, the peptide treatment decreased the heart rate and peak left ventricular pressure and delayed relaxation in both basal and after isoproterenol stimulation [18]. These contradicting reports are based on the global disruption of PKA anchoring, but to identify the role of AKAPs in calcium cycling and contraction, knockouts of individual AKAPs are required.

## 3. AKAP Regulating L-type Calcium Current

AKAP7α: AKAP7 has four alternatively spliced isoforms (α, β, γ, and δ), and all four isoforms harbor the PKA-binding domain. AKAP7α (AKAP15 or AKAP18), the shortest isoform of AKAP7, has N-terminal lipid modifications (myristoylation and palmitoylation) that help AKAP7α to be expressed in the inner leaflet of the plasma membrane, where it localizes with Cav1.2 and facilitates the augmentation of the calcium current in a heterologous system [19]. Later, another study identified that AKAP7α localizes in transverse tubules of the skeletal muscle cells and co-localizes with Cav1.2 [20]. AKAP7α binds to the distal c-terminus of the LTCC with its leucine zipper-like motif. Later, in ventricular cardiac myocytes, AKAP7α was identified to facilitate the interaction between PKA and Cav1.2, and by using the synthetic peptides that disrupt AKAP7 from Cav1.2, it was efficiently shown that AKAP7α is indispensable for the increased calcium current following β-adrenergic stimulation [21]. Furthermore, by using 1. a peptide-based blocker [20]; 2. preventing AKAPα membrane expression [19]; 3. the disruption of the leucine-zipper-like motif in AKAP7α [21]; and 4. the deletion of AKAP7 binding domain in Cav1.2 provided enough evidence that AKAP7α is responsible for the augmentation of L-type calcium currents in cardiac myocytes [22].

Though several phosphorylation sites were reported in both the α and β subunits of Cav1.2, none really affected the β-adrenergic stimulation-enhanced calcium current and calcium transients in mouse cardiac myocytes. The recent identification of Rad G-protein, a calcium channel inhibitor, as a PKA substrate answered the long-standing question of how the β-adrenergic receptor augments voltage-gated calcium current [7]. It is important to highlight that Rad phosphorylation is indispensable for the β-adrenergic reserve, as mice with Rad mutants that cannot be phosphorylated have a near complete attenuation of β-adrenergic response [9]. Whether AKAPα plays a role in Rad phosphorylation is not yet known.

## 4. AKAPs Regulating Calcium Reuptake into SR

**AKAP7δ:** AKAP7δ is the longest and the second AKAP7 isoform that is known to affect calcium cycling. A comprehensive study on the evolutionary aspects of AKAP7 revealed that AKAP7δ is the only long isoform in rats, whereas AKAP7γ is the predominantly expressed isoform in both mice and humans. Interestingly, both AKAP7γ (in mice) and AKAP7δ (in rats) were found to be expressed in SR [23]. AKAP7δ isoform was found to be co-immunoprecipitated with SERCA2a/PLN complex. Using a peptide-based approach to disrupt PLN from AKAP7δ and siRNA knock-down approaches, the influence of AKAP7δ in PLN phosphorylation and calcium reuptake was demonstrated. However, the experiments to show PLN phosphorylation/calcium reuptake were performed in immature neonatal rat cardiac myocytes, where the cAMP/PKA compartmentation can be vastly different from matured adult myocytes [24]. Later in 2012, a global AKAP7 knockout mouse was generated, and this mouse was expected to perform poorly on cardiac calcium cycling as both the α and δ isoforms of AKAP7 known to regulate calcium transients were deleted. Surprisingly, the phosphorylation of Cav1.2 and PLN were unaffected, and hence the calcium currents and reuptake were normal and comparable to the wildtype [25]. The normal calcium cycling in AKAP7 knockout raised a few questions. 1. Is there another AKAP that compensates for the loss of AKAP7 isoforms in mice? 2. Are different pools of PLN and Cav1.2 regulated by multiple AKAPs including AKAP7?

Though the AKAP7δ isoform does not have a membrane-targeting domain or N-terminal lipid modifications like AKAP7α, it can be identified in the SR [24] and aquaporin-2-bearing vesicles. An unconventional mechanism of membrane targeting is possible through the arrangement or distribution of positively charged amino acids in its sequence that form a binding surface for negatively charged lipid moieties in the bilayer [26]. Whether AKAP7δ uses an adapter protein or binds directly to PKA substrates such as PLN or RyR2 in cardiac myocytes and aquaporin-2 in renal principal cells must be investigated.

Moreover, two fascinating features of AKAP7 were unraveled from the studies which revealed that they are highly mobile [27] and able to dissociate and associate from phosphorylated and dephosphorylated PLN, respectively [28]. These studies led us to answer how AKAPs in a nanomolar range were able to mediate the phosphorylation of PLN, which is in the micromolar range.

**AKAP7 and phosphatase:** AKAP7 mediates the inhibition of Ser/Thr-specific protein phosphatase 1 (PP1) by phosphorylating the PP1 inhibitor, I-1 [29]. It is noteworthy to mention that I-1 phosphorylation is reduced in human heart failure [30]. In mice, the overexpression of active (constitutively phosphorylated) I-1 increases PLN phosphorylation and cardiac contractility not only in healthy subjects but also prevents hypertrophy and heart failure in mice with transverse-aortic constriction [31]. During β-adrenergic stimulation, the inhibition of PP1 by PKA is achieved by the phosphorylation of I-1 at Thr35 [32]. PP1 and I-1 are in complex with the AKAP7δ isoform in rat hearts, and the ablation of AKAP7δ prevents the I-1 phosphorylation and inhibition of PP1 [29].

**AKAP7 and CaMKIIδ:** Recently, AKAP7δ was identified to anchor CaMKIIδ close to both PLN and RyR2 in rat hearts. AKAP7δ has bidirectional and opposing roles in regulating CaMKIIδ activity. The N-terminal region of AKAP7δ inhibits CaMKIIδ-mediated PLN phosphorylation at Thr17 in both in vitro and in vivo experiments and delays the calcium reuptake in cardiac myocytes paced at higher frequencies, whereas the c-terminal region in the AKAP7δ, which is homologous to neuronal CaMKIIδ activator peptide, acts vice versa. RyR2 is phosphorylated by AKAP7δ-bound CaMKIIδ at Ser2814, and the phosphorylation increased the occurrence of calcium sparks [33,34].

**AKAP7 and PDE:** AKAP7δ was identified to bind PDE4D3 in renal collecting duct principal cells. AKAP7δ mediates the PKA phosphorylation and membrane expression of the osmotic water permeability channel, aquaporin-2. After membrane expression, the water channel activity was downregulated by PDE4D3 phosphorylated by PKA bound to AKAP7δ [35]. In cardiac myocytes, cAMP in the PLN compartment is regulated by PDE4D [36], which is probably confined to AKAP7δ.

**mAKAPβ and RyR2 phosphorylation:** mAKAPβ, the muscle-specific AKAP (AKAP6 or AKAP100) was first identified in the perinuclear and SR fractions of H9c2 cells [37]. In adult rat cardiac myocytes, mAKAPβ was co-localized and co-immunoprecipitated with RyR2 in the nuclear envelope [38]. The adapter or scaffold proteins for PKA-RIIα (mAKAPβ) and the phosphatases PP1 (Spinophilin) and PP2A (PR130) were identified to bind the leucine-zipper motifs in the channel [39]. mAKAPβ mediates the phosphorylation of RyR2 by PKA [39]. The PKA-mediated phosphorylation of RyR2 at Ser2808 (S2809 in rabbits) [40] results in channel instability and increased sensitivity to calcium-dependent activation due to the dissociation of FKBP12.6/calstabin 2 [10]. FKBP12.6 mediates the coupled-gating of RyR2 channels and is critical for RyR2 activation that is not associated with Cav1.2 [41]. The theory of FKBP12.6 dissociation from phosphorylated RyR2 has been challenged by other investigators [42]. In heart failure patients and failing canine hearts, the Ser2808 was found to be hyperphosphorylated due to the loss of phosphatase activity in the RyR2 complex [10]. The mechanistic role of RyR2 hyperphosphorylation in heart failure and arrhythmia remains controversial [43]. AKAP7δ-bound CaMKIIδ also phosphorylates RyR2 at the Ser2814 [34]. However, there is no conclusive evidence to show that mAKAPβ regulates RyR2 phosphorylation or calcium release from SR.

**PDE and phosphatase regulation by mAKAPβ:** cAMP levels in the mAKAPβ complex are negatively regulated by PDE4D3. The available pool of PDE4 in this complex accounts for around 5% of the total PDE4 activity in the myocardium. The tonic activity of this PDE4D3 pool maintains PKA in a quiescent state. The β-adrenergic stimulation-induced phosphorylation of PDE4D3 increases PDE activity several-fold [44]. The ablation of PDE4D in mice results in a loss of PDE activity in the RyR2 compartment, the hyper-phosphorylation of Ser2808, and a subsequent increase in channel open probability. PDE4D knockouts display age-related cardiomyopathy and increased arrhythmia susceptibility. In agreement with the mouse data, RyR2 associated-PDE4D levels are reduced in heart failure patients [45]. Another study identified increased basal contractility in PDE4D knockouts, but this phenotype is attributed to increased basal PLN phosphorylation [36]. Spectrin-repeat-like sequences in mAKAPβ facilitate nuclear membrane targeting [46], and nesprin 1α, a nuclear transmembrane protein, serves as an adapter for mAKAPβ [47].

The phosphatase calcineurin associated with mAKAPβ is critical for catecholamine-induced cardiomyocyte hypertrophy. The expression of an mAKAPβ mutant that lacks a calcineurin binding domain or peptide-based blocking of mAKAP-calcineurin interaction reduced the hypertrophic response in vitro [48,49]. The perinuclear calcium microdomain was identified to be responsible for the calcineurin activation and the subsequent NFAT translocation and cellular hypertrophy. The perinuclear calcium increase is dependent on the mAKAPβ-regulated increase in the pSer2808 RyR2 [50]. Recently, mAKAPβ has been reported to be associated also with PLN in both transfected HEK293 cells and neonatal mouse cardiac myocytes. mAKAPβ/PKA signaling cascade activation increased SERCA2a activity in PLN-overexpressed HEK293 cells. In adult rat cardiac myocytes, PLN was immunoprecipitated with mAKAPβ. Microscopic evaluation revealed PLN/mAKAPβ co-localization in the perinuclear region of adult rat cardiac myocytes. Like AKAP7, mAKAPβ binding to PLN depends on its phosphorylation status [51].

## 5. Role of AKAP5 in Sympathetic Stimulation

AKAP5, or AKAP150, the murine ortholog of AKAP79, is another classic example of the bidirectional role that AKAPs play in regulating protein phosphorylation states. AKAP5 binds the kinases, PKA, PKC [52], and phosphatase calcineurin. Surprisingly, binding to AKAP5 inhibits calcineurin activity [53]. AKAP5 is expressed in T-tubules and regulates calcium transients in adult mouse cardiac myocytes. Unexpectedly, the expression of mutant AKAP5, which cannot bind PKA, did not affect the calcium transient. Hence, other proteins that bind AKAP5-like AC6 and calcineurin are involved in AKAP5-regulated calcium transient. Cav1.2 in caveolin-rich compartments is phosphorylated, and this mechanism is important for the elicitation of calcium transient. However, in AKAP5 knockout cardiac myocytes, Cav1.2 in caveolin-rich compartments was not phosphorylated after adrenergic stimulation. Interestingly, the phosphorylation of PLN and RyR2 are inhibited too [54]. In rat brains, AKAP5-bound PKA mediates the negative feedback inhibition on AC5/6 [55]. In the same system, AKAP5 was identified to bind and phosphorylate β_1_ [56] and β_2_ [57] adrenergic receptors. The PKA-mediated phosphorylation of β_1_ adrenergic receptors (at Ser213) is critical for receptor recycling and resensitization [56]. For AMPA-type glutamate receptor activation and synaptic plasticity, AKAP5-AC6 association, but not AKAP5-PKA, is critical [58]. AKAP5 knockout depleted PKA from Cav1.2 and reduced its phosphorylation in neurons [59]. Contrary to previous findings that RyR2 is not associated with AKAP5 [54], RyR2 and PLN were reported to be co-immunoprecipitated with AKAP5 [60].

## 6. Other Cardiac AKAPs Affecting Calcium Cycling and Sympathetic Activation

Gravin (AKAP12 or AKAP250) binds β_2_-adrenergic receptors in addition to PKA, PKC, and PP2B. Gravin, like AKAP7, possesses positively charged domains that electrostatically stick to the submembrane compartments from where it mediates the PKA/PKC-dependent β_2_-adrenergic receptor sequestration and resensitization. These positively charged domains can bind calmodulin at high calcium concentrations and hence regulate the receptor recycling process [61,62]. In transgenic mice with non-functional gravin, the basal and isoproterenol-augmented contractility is increased, which is in line with faster calcium transient, decreased phosphorylation of PKA-mediated β_2_-adrenergic receptors, and desensitization. This leads to increased β_2_-receptors’ availability at the cell membrane. However, the phosphorylation of both PLN and RyR2 is unaffected [63]. Hence, targeting gravin is proposed as a therapy for heart failure.

Phosphoinositide 3-Kinase γ (PI3Kγ) acts as an AKAP and regulates β_2_-adrenergic signaling. Challenged PI3Kγ knockouts are prone to ventricular tachycardia due to the loss of PDE3 and PDE4A/B compartmentation and abnormal cAMP levels leading to the hyperphosphorylation of Cav1.2 and PLN. This leads to increased calcium amplitude and spark occurrence after adrenergic stimulation resulting in the arrhythmic phenotype [64,65].

## 7. Calcium Regulating AKAPs in Heart Diseases

**Figure 2:**
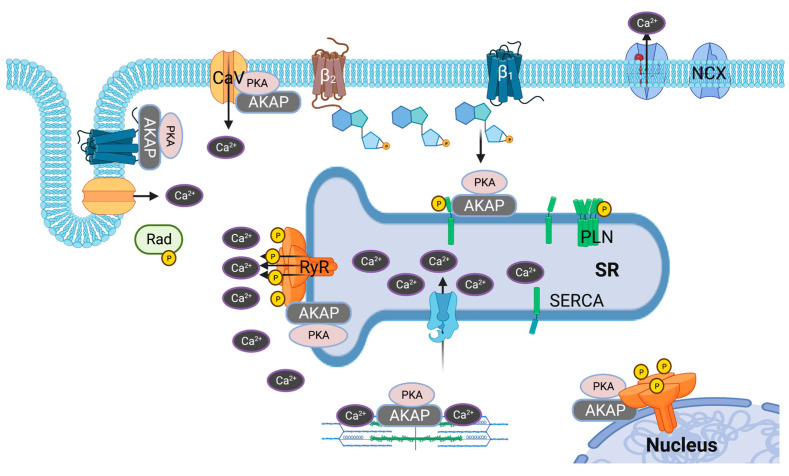
Dysregulation of calcium cycling in heart failure: During heart failure, calcium cycling is affected due to the loss of T-tubules and reduced expression/activity of SERCA2a. RyR2 is hyperphosphorylated leading to increased calcium sparks. Illustrations in Figure 2 are created with BioRender.com.

In the pathophysiology of heart disease, calcium cycling is dysregulated due to: 1. the loss of T-tubules, thereby disturbing the CICR from SR [66]; 2. reduced SERCA2a expression/activity leading to reduced capacity for calcium reuptake [67]; and 3. increased RyR2 phosphorylation resulting in increased calcium sparks [10]. cAMP compartmentation in these calcium-handling proteins is impaired due to the redistribution of β-adrenergic receptors [68,69] and PDEs [70]. Only limited data are available on the role of AKAPs in the pathophysiology of heart diseases (Figure 2).

Cardiomyocyte-specific mAKAPβ knockout is protective against pressure overload-induced hypertrophy and heart failure. The ablation of mAKAPβ prevented myocardial apoptosis, fibrosis, left atrial hypertrophy, and pulmonary edema [71]. Mechanistically, mAKAPβ-dependent calcium signaling/calcineurin activation and NFAT translocation, and the phosphorylation of class IIa Histone Deacetylase (HDAC) by PKA or protein kinase D (PKD), promote pathological hypertrophy [72]. Recently, mAKAPβ was identified to facilitate the phosphorylation of serum response factor (SRF) at Ser103 by p90 ribosomal S6 Kinase Type 3 (RSK). The adeno-associated virus (AAV)-mediated expression of peptides to uncouple either RSK3 or PP2A from mAKAPβ improves cardiac function and inhibits concentric (blocking RSK3) and eccentric (blocking PP2A) hypertrophy and remodeling [73].

AKAP5 is reported to be involved in pathological remodeling and the loss of contractile reserves in the pathophysiology of heart failure. Three-month-old AKAP5 global knockout mice developed hypertrophy, ventricular dilatation, increased fibrosis, and systolic dysfunction that progressed with age. Interestingly the beta-blocker carvedilol recovered the hypertrophy and systolic dysfunctions in AKAP5 knockouts by a yet unidentified mechanism. AKAP5 knockout mice subjected to chronic isoproterenol infusion did not suffer badly in hemodynamics compared to wildtype due to the enhanced activity of both calcineurin and CaMKII. The ablation of AKAP5 affected β1-adrenergic receptor recycling, due to the loss of calcineurin from the β1 compartment [74]. In pressure overload-induced hypertrophy, the AKAP150 levels are reduced by 50%, indicating a protective role for this AKAP in the heart [60]. Similarly, when the H9C2 cardiac myocyte cell line was exposed to hypoxia and reoxygenation, AKAP150 expression was reduced [75]. The cardiomyocyte-specific deletion of AKAP150 predisposes mice to dilated cardiomyopathy and cardiac dysfunction. An increase in the phosphorylation of both RyR2 and PLN is inhibited after hypertrophy or isoproterenol stimulation in the knockouts. Calcium transient is also affected in hypertrophied AKAP5 knockouts [60].

The long QT syndrome (LQT8) in Timothy patients due to a gain-of-function mutation G406R in a cytoplasmic loop of Cav1.2 results in the slow inactivation of L-type calcium channels and frequent arrhythmias. As AKAP150 was found to be abnormally coupled to LQT8 and increases the chance for coupled gating and the open probability of the channel, the ablation of AKAP150 was considered a potential therapy. The deletion of AKAP150 in mouse models with LQT8 abolished the delay in the calcium current inactivation, hence preventing the long QT syndrome and arrhythmias [76].

## 8. Therapeutic Targeting of AKAP to Improve Cardiac Function

In human heart failure and animal models with induced heart failure, calcium handling is severely affected either by structural alterations in the dyadic cleft or altered levels of calcium-handling proteins. In heart failure patients with dilated cardiomyopathy, SERCA2a levels are reduced by 28% when normalized to the PLN levels leading to insufficient calcium reuptake capacity [67]. Though gene therapy to express SERCA2a improved cardiac output in sheep and guinea pigs with heart failure [77,78], a phase IIb randomized clinical trial (CUPID2) in heart failure patients with reduced ejection fractions did not meet the expected patient outcome [79]. Hence, new targeted therapies to improve SERCA2a activity such as strategies to enhance the phosphorylation of PLN by AAV-mediated expression of AKAPs could be an attractive option.

In the last few years, reducing global cAMP levels [80] and PKA activity [81] by AAV9-mediated PDE expression and protein kinase inhibitor (PKI), respectively, are considered as viable and more targeted alternatives for beta-blockers. Recently, PDE3 gain-of-function mutants in rats are shown to be protective against heart failure despite being hypertensive. Although the β-adrenergic response was normal, PLN phosphorylation was reduced in the mutant rats, indicating altered calcium reuptake kinetics [82]. Although these data imply that reducing cAMP or PKA activity is a better therapeutic model than beta-blockers, reducing global cAMP/PKA can severely affect cardiac contractility. Hence, a more selective shRNA knockdown approach to target AKAPs such as AKAP6 that promote hypertrophy or the AAV9-mediated expression of AKAPs to enhance PLN and/or Rad phosphorylation is a tailor-made option to improve calcium kinetics and contraction in the failing heart.

Peptide disruptors to uncouple PKA, PDE, or phosphatase from the AKAP signaling complex can modulate substrate phosphorylation. Peptides derived from AKAP7 are used as disruptors of the AKAP/PKA signaling complex and can be used to modulate substrate phosphorylation. AKAP7 peptides to uncouple PDEs can be used to increase PLN phosphorylation. Hence, there are several plausible methods for targeted therapies based on AKAP-signaling complexes.

## 9. Conclusions

Calcium cycling directly affects cardiac function, and any alterations in the time kinetics and amplitude of calcium cycling are detrimental. cAMP/PKA compartmentation mediated by AKAPs in different microdomains is responsible for cardiac function during the fight-or-flight response. In this review, we tried to elaborate on the findings about the AKAPs that regulate calcium cycling in cardiac myocytes. However, there remain several unanswered questions. 1. What are the AKAPs that regulate PLN, SR-bound RyR2, and Rad phosphorylations? 2. Can multiple AKAPs regulate the phosphorylation of the same protein in different microdomains in the same cell? 3. How are AKAPs affected in a failing heart with massive changes in membrane structures and compartmentation? 4. Finally, do we know all the AKAPs, or are we yet to identify new AKAPs or old AKAPs with new functions? To answer these questions, we need cardiomyocyte-specific knockouts of several AKAPs to dissect the AKAP-selective cardiac function in both health and disease. Once the AKAP-specific function has been established, a tailor-made therapy to improve calcium cycling and related cardiac function can be established.

Classical β-blockers used to blunt the beta-adrenergic overdrive in heart failure patients affect not only global but also microdomain cAMP/PKA activity that is required for cardiac contractility. Targeting pro-hypertrophic AKAPs with AAV9-mediated cardiac-specific knockdown or an over-expression of beneficial AKAPs to modulate cAMP/PKA activity in microdomains is a more selective therapeutic approach. For instance, the AAV-mediated expression of PLN-binding AKAPs or PKA-RII that preferentially binds AKAP7 [83] can be a potential alternative treatment to increase SERCA activity in heart failure patients. Additionally, peptide mimetics to selectively dislocate specific signaling molecules such as kinase or phosphatase from pro-hypertrophic AKAPs such as mAKAP is a more appropriate treatment strategy to prevent cardiac remodeling triggered by sympa-thetic overdrive. Finally, we propose that more preclinical studies need to be performed to validate the peptide and AAV-based approaches to target AKAPs in calcium-handling microdomains to treat heart failure in translational models.
